# Green Extraction of Tea Polysaccharides Using Ultrasonic-Assisted Deep Eutectic Solvents and an Analysis of Their Physicochemical and Antioxidant Properties

**DOI:** 10.3390/foods14152601

**Published:** 2025-07-24

**Authors:** Haofeng Gu, Lei Liang, Yang Wei, Jiahao Wang, Yibo Ma, Jiaxin Shi, Bao Li

**Affiliations:** 1 School of Modern Agriculture & Biotechnology, AnKang University, Ankang 725000, China; wjh18292921725@163.com (J.W.); 18098040514@163.com (Y.M.); x3235655039@163.com (J.S.); 17789269938@163.com (B.L.); 2 Shaanxi Provincial-Municipal Jointly Built Key Laboratory of Tea, AnKang University, Ankang 725000, China; 3 Department of Food Science and Engineering, School of Agriculture and Biology, Shanghai Jiao Tong University, Shanghai 200240, China; sunny1994@sjtu.edu.cn

**Keywords:** deep eutectic solvents, polysaccharides, ultrasonics, tea, oxidative stress

## Abstract

In this study, the ultrasonic-assisted extraction of deep eutectic solvents (UADES) for tea polysaccharides was optimized, and their physicochemical properties and antioxidant activities were analyzed. The optimal DES comprised choline chloride (CC) and ethylene glycol (EG) in a molar ratio of 1:3, with a water content of 40%. The optimized condition was an extraction temperature of 61 °C, an ultrasonic power of 480 W, and an extraction time of 60 min. The UADES extraction rate of polysaccharides (ERP) was 15.89 ± 0.13%, significantly exceeding that of hot water (HW) extraction. The polysaccharide content in the UADES-extracted tea polysaccharides (UADESTPs) was comparable to that of hot-water-extracted tea polysaccharides (HWTPs) (75.47 ± 1.35% vs. 74.08 ± 2.51%); the UADESTPs contained more uronic acid (8.35 ± 0.26%) and less protein (12.91%) than HWTP. Most of the UADESTPs (88.87%) had molecular weights (Mw) below 1.80 × 10^3^ Da. The UADESTPs contained trehalose, glucuronic acid, galactose, xylose, and glucose, with molar ratios of 8:16:1:10. The free radical scavenging rate and total reducing power of the UADESTPs were markedly superior to those of the HWTPs. Moreover, the UADESTPs had a better alleviating effect on H_2_O_2_-induced oxidative injury in HepG2 cells. This study develops an eco-friendly and efficient extraction method for tea polysaccharides, offering new insights for the development of tea polysaccharides.

## 1. Introduction

Tea is the most widely consumed plant-based beverage globally, which has spread to over 160 countries. Owing to its outstanding physiological functions, tea has been increasingly utilized in the medical and healthcare industries. Polysaccharides are an important component of tea. Plenty of studies have confirmed that tea polysaccharides possess various bioactive properties, including the alleviation of diabetes [1], antioxidant activity [2], enhanced immune function [2], and the regulation of gut health [1]. This has led to increased interest in their extraction and development in recent years.

HW extraction is a traditional method for extracting tea polysaccharides; however, it has several drawbacks, including prolonged extraction times, high energy consumption, and low extraction efficiency [3]. To enhance extraction efficiency, enzymes, ultrasound and microwaves have been applied in the extraction process [3]. From an industrial production perspective, ultrasonic-assisted extraction presents broader application prospects [4]. The method primarily leverages the cavitation effect of ultrasound to effectively disrupt cell walls and facilitate the release of intracellular polysaccharides [4]. Ultrasound can also partially cleave glycosidic bonds within polysaccharides, thereby reducing their Mw and enhancing their solubility and biological activity [5]. This technique has already been applied to the extraction of plant polysaccharides [4,6]. However, the method is rarely used in the extraction of tea polysaccharides.

Additionally, in the current context that emphasizes green and eco-friendly extraction, green methods with high extraction efficiency are garnering increasing attention. DES, composed of hydrogen bond donors (HBDs) and hydrogen acceptors (HBAs), are emerging as novel sustainable and efficient extraction solvents with low or negligible toxicity [7]. Due to their high extraction ability, DES has recently been employed in polysaccharide extraction. For instance, the efficiency of wolfberry polysaccharide extraction using DES is 2.87-fold higher than that of HW [8]. However, the extraction time of DES is still long, indicating that this extraction strategy necessitates further optimization [9]. Ultrasonic-assisted extraction can significantly reduce extraction times. Moreover, combining multiple extraction strategies can further improve the extraction efficiency of polysaccharides [3]. However, to date, the UADES extraction of tea polysaccharides has not been extensively reported. This technology requires focused investigation.

Therefore, this study aimed to optimize the UADES extraction for green tea polysaccharides. Then, the physicochemical properties and structural characterization were investigated, along with the anti-oxidant activity of the extracted tea polysaccharides. This research offers a technical reference for developing efficient and environmentally sustainable extraction methods for tea polysaccharides.

## 2. Materials and Methods

### 2.1. Materials

Green tea was brought from Ziyang Qin Selenium Ecological Agriculture Co., Ltd., Ankang, China. Choline chloride (CC), lactic acid (LA), oxalic acid (OA), ethylene glycol (EG) and 1,4-butanediol were bought from Xi’an Tai’an experimental re-agent Co., Ltd., Xi’an, China. DMEM medium, CCK 8 kit, catalase (CAT) kit, glutathione peroxidase (GSH-Px) kit and DCFH-DA fluorescent probe, FBS and HepG2 were purchased from Beina Chuanglian Bio-technology Co., Ltd., Xinyang, China.

### 2.2. DES System Preparation

DES systems were prepared using the heating method [8]. Briefly, HBAs were mixed with different HBDs and stirred (60 °C) to obtain transparent solutions; the molar ratio and the water contents in the DES systems are listed in Table 1.

### 2.3. Selection of DES and Molar Ratio of HBA to HBD in DES System

Five grams of tea powder was mixed with different DES at a ratio of 1:30 (g:mL). Tea polysaccharides were ultrasonic-assisted extracted at 50 °C (60 min, 400 W) using an ultrasonic extractor (SCIENTZ-500C, SCIENTZ Biotechnology, Ninbo, China), followed by centrifugation (2000× *g*, 10 min). After concentrating the supernatant tenfold using a rotary evaporator, tea polysaccharides were precipitated (4 °C, 12 h) by adding four times the volume of anhydrous ethanol. The precipitates were collected and a Sevag reagent was used to remove proteins [10], followed by dialysis in deionized water (MWCO 300 Da) for 48 h to remove small molecules. The dialysis solution was collected, and the UADESTPs were obtained after vacuum freeze-drying. Polysaccharide contents were measured [10]. The ERP was calculated according to Formula (1), and the DES exhibiting the maximum EPR value was selected for further analysis:

(1) $$\mathrm{ERP}\;(\%)\;=\;\frac{m}{M}\times 100$$

m and M stand for the weight of extracted tea polysaccharides and raw tea powder, respectively.

After that, the molar ratio (1:1–1:5) of HBAs to HBDs in the DES system was further optimized and the one with the highest EPR was selected.

### 2.4. Optimization of UADES Extraction

#### 2.4.1. Single-Factor Experiments

Briefly, the effect of four factors on the EPR of UADES extraction was evaluated: water content (10–50%), extraction temperature (40–80 °C), extraction time (20–100 min) and ultrasonic power (400–720 W). Each factor was evaluated while the others were fixed.

#### 2.4.2. Box–Behnken Design (BBD) Experiments

Briefly, the independent variables were water content (A), temperature (B) and extraction time (C), and EPR was used as the dependent variable (Table 2). The BBD test design is listed in Table 2. The results were fitted using the second-order polynomial model [10]:

(2) $$Y=\;\beta 0\;+\;\sum_{i=0}^{3}\beta_{i}X_{i}+\;\sum_{j=0}^{3}\beta_{ii}X_{i}^{2}+\;\sum \sum_{i=0}^{3}\sum_{j=0}^{3}\beta_{ij}X_{i}X_{j}$$

where Y is the dependent variable, X_i_ and X_j_ are different variables, and β_0_, β_i_, β_ii_, and β_ij_ are regression coefficients for intercept, linearity, square, and interaction, respectively.

### 2.5. Determination of Proteins, Uronic Acid, and Mw of Tea Polysaccharides

The protein content was determined using the *Kjeldahl* method, and uronic acid content was measured using the m-hydroxybiphenyl method with D-glucuronic acid as the standard [11].

The Mw of tea polysaccharides was analyzed via HPGPC using a TSK-Gel G4000PW analytical column (7.8 mm × 300 mm) (Tosoh Corporation, Yamaguchi shi, Japan) at 35 °C [10]. The mobile phase consisted of 0.1 mol/L Na_2_SO_4_ solution with a flow rate of 0.5 mL/min and an injection volume of 20 μL.

### 2.6. HW Extraction of Tea Polysaccharides

The HW extraction of tea polysaccharides was conducted according to a method reported previously [12]. Briefly, tea powder (10 g) was mixed with distilled water (200 mL) and extracted at 55 °C for 3 h. The mixture was centrifuged (2000× *g*, 10 min) to collect the supernatant. The crude tea polysaccharides were precipitated using 95% ethanol, and deproteinization was carried out with a Sevag reagent. The collected sample was dialyzed using deionized water (MWCO 300 Da). After lyophilization, HWTP was obtained.

### 2.7. Charicterization of Tea Polysaccharides

The micro-structure of tea polysaccharides was observed using a scanning electron microscope (SEM). Briefly, samples (1 mg) were sprayed with gold and the morphologies were analyzed using an SEM (Geminisigma 300, ZEISS, Oberkochen, Germany). The accelerating voltage was set at 3 kV, and the magnification was 1000×.

The monosaccharide composition of tea polysaccharides was measured using the PMP pre-column derivatization method reported previously [13]. Standard monosaccharides (trehalose, mannose, ribose, rhamnose, glucuronic acid, galacturonic acid, glucose, galactose and xylose) were used as a reference.

The ultraviolet–visible (UV) spectra (190 to 400 nm) of tea polysaccharides (1 mg/mL) were analyzed using a UV spectrophotometer (UV-3200S, MAPADA, Shanghai, China). The Fourier transform infrared spectroscopy (FT-IR) of tea polysaccharides was analyzed using an FT-IR (IFS-55, Bruker, Saarbrücken, Germany) from 4000 cm^−1^ to 400 cm^−1^.

### 2.8. Anti-Oxidant Activity Assay

The DPPH, ABTS^+^, OH·scavenging rate, and reduction capability of different concentrations (0.16, 0.32, 0.48, 0.64 and 0.8 mg/mL) of samples were measured according to the methods reported previously [10,14]. Distilled water and Vc were used as the blank control and positive control, respectively.

### 2.9. Cell Culture and Cell Viability

HepG2 cells were cultured in a DMEM medium supplemented with FBS (10%, *v*/*v*) and penicillin–streptomycin (1%, *v*/*v*) at 37 °C in an incubator with 5% CO_2_. Cells were seeded in 96-well plates. After 24 h, the cells were then treated with different concentrations (0, 200, 400, 800 and 1600 μg/mL) of samples (UADESTP or HWTP) for 24 h, and the cell viability was measured using CCK 8 kits.

### 2.10. Protective Effect on H_2_O_2_-Induced Oxidative Injury Assay

The assay was conducted following a previously described method [15]. Briefly, the experiment was composed of four groups: the control group, model group, UADESTP group, and HWTP group. Cells in the control group were cultured under standard conditions. Cell viability in each group was assessed using the CCK8 kit, and the activities of SOD, GSH-Px, and CAT were measured following the kit instructions. Intracellular reactive oxygen species (ROS) were labeled with the DCFH-DA fluorescent probe following the kit instructions, and the fluorescence intensity of cells in each group was measured. The percentage of fluorescence intensity relative to the control group represents the amount of intracellular ROS.

### 2.11. Statistical Analysis

Data are presented as the mean ± SD. Statistical significance was assessed using SPSS 16.0 (IBM, NewYork, NY, USA). The Duncan test and the analysis of variance (ANOVA) were used to analyze the data and determine significant differences at the level of *p* < 0.05.

## 3. Results and Discussion

### 3.1. Effect of DES Type and the Ratio of HBAs to HBDs on EPR

**Figure 1:**
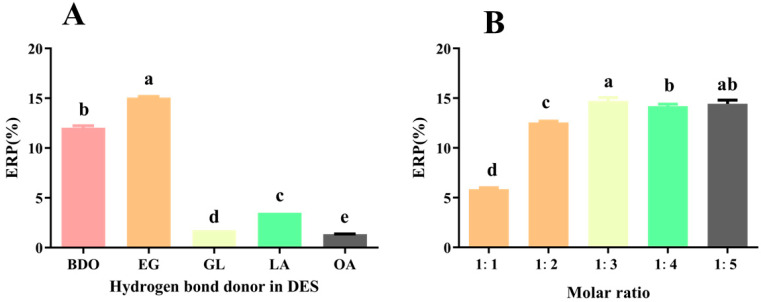
(**A**) Effect of different DES on ERP. (**B**) Effect of different molar ratios of EG to choline chloride in DES on ERP. Different letters indicate significant differences (*p* < 0.05), and the same letters represent no significant difference (*p* > 0.05).

Choline chloride (CC) is highly biodegradable and cost-effective, so it is commonly used as an HBA to prepare DES for extracting plant polysaccharides [7]. Herein, DES was prepared using CC as the HBA. As shown in Figure 1A, the DES (CCEG) with ethylene glycol (EG) as the HBD exhibited the highest ERP (15.05 ± 0.12%), significantly exceeding that of other groups (*p* < 0.05). This difference might be caused by variations in diffusivity, solubility, viscosity, and polarity among the different DESs. Studies have shown that the polarity of the DES is positively correlated with its polysaccharide solubility, and the polarity of EG is higher than that of other HBDs [16], which may explain the superior extraction efficiency of the CCEG. Additionally, EG has a relatively low viscosity (26 mPa·s at 20 °C), which results in the better fluidity of the CCEG, facilitating the dissolution and extraction of polysaccharides. Therefore, CCEG was selected for the ultrasonic-assisted extraction of tea polysaccharides.

Figure 1B shows that, as the ratio of HBA to HBD decreases, the ERP initially increases and then decreases, peaking at 15.72 ± 0.34% when the ratio is 1:3. This suggests that the acceptor-to-donor ratio in DES significantly influences the ERP. The ratio directly influences the solution’s polarity and viscosity. When the ratio of HBD to acceptors reaches its optimal level, the interaction forces between them tends to saturate [17]. Further increasing the ratio decreases the solvent’s fluidity and increases its viscosity, thereby reducing the efficiency of polysaccharide extraction [18]. A similar phenomenon was observed in the study conducted by Qu et al., where a DES prepared using CC and EG was applied to extract abalone polysaccharides [19]. Therefore, the ratio of CC to CE (1:3) was chosen in the following study.

### 3.2. Effect of the Water Content of DES and the Extraction Temperature, Extracting Time and Ultrasonic Power on ERP

**Figure 2:**
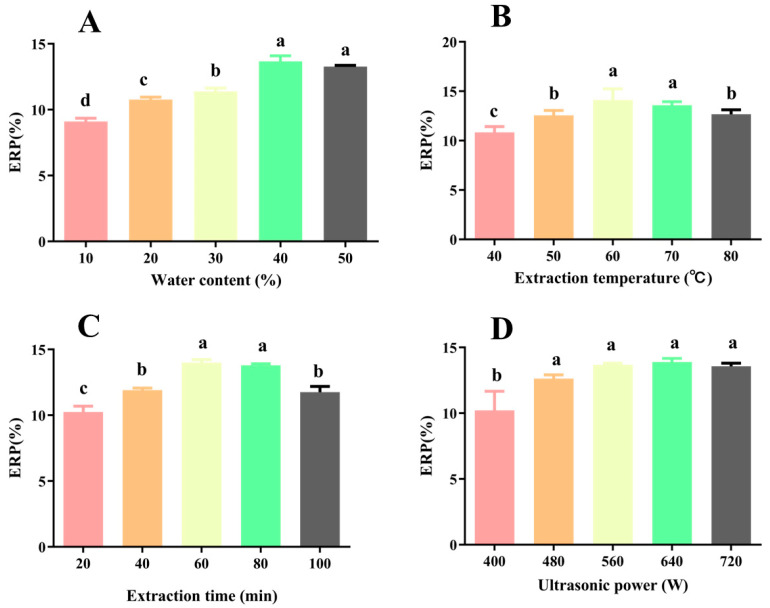
Effects of (**A**) water content, (**B**) extraction temperature, (**C**) extraction time, and (**D**) ultrasonic power on EPR. Different letters indicate significant difference (*p* < 0.05), and the same letters represent no significant difference (*p* > 0.05).

Figure 2A shows that ERP increases significantly with rising water content, peaking at a water content of 40%. A suitable water content reduces DES viscosity, thereby improving the mass transfer rate between the polysaccharide and solvent. Excessive water content, however, can disrupt the hydrogen bonds between CC and EG, reducing its interaction with polysaccharide components, and ultimately hindering polysaccharide extraction [20].

Figure 2B indicates that, as the temperature increases, ERP first increases and then decreases. The ERP reaches its peak (13.91 ± 1.49%) at 60 °C. As the temperature increases, the movement of solvent molecules accelerates, enhancing the contact between intracellular polysaccharides and the solvent, which facilitates polysaccharide extraction. Additionally, as the temperature increases, the viscosity of DES decreases [21]. However, further temperature increases cause polysaccharide degradation, reducing the extraction rate [7]. Similar trends were reported in the extraction of *Indocalamus tessellatus* leaf polysaccharides using DES [21].

Figure 2C demonstrates that the extraction time significantly impacts EPR. Within the range of 20 to 80 min, EPR gradually increases; however, the EPR significantly decreases with prolonged extracting times. Moreover, there is no significant difference in EPR between 60 and 80 min. Similarly, in a study of the UADES extraction of bamboo leaf polysaccharides, 60 min was also identified as the optimal extraction time [22].

Figure 2D shows that, as the ultrasound power increases from 400 W to 480 W, the ERP significantly rises. Increasing the ultrasonic power enhances cavitation effects, thereby promoting polysaccharide extraction. However, further increases in power do not significantly affect the ERP. Excessive ultrasound power not only wastes energy but also leads to the structural degradation of polysaccharides [23]. Therefore, 480 W is chosen in the following studies.

### 3.3. Response Surface Optimization

Based on the above results, three factors including (A) water content (30–50%), (B) temperature (50–70 °C) and (C) extraction time (40–80 min) were selected in BBD, and the results are listed in Table 2. After multiple regression analysis, the second-order regression equation for ERP was derived as follows: Y = 15.15 + 0.015A + 0.1B − 8.24 × 10^−3^C + − 0.047AB + 0.036AC − 0.027BC − 1.04A^2^ − 0.71B^2^ − 1.10C^2^. As indicated in Table 3, the *p*-value of this model is below 0.001, whereas the *p*-value for lack of fit is 0.549, exceeding 0.05. The R^2^ and adjusted R^2^ values are both close to 1. These results suggest that the established regression model is significant and fits well across the entire regression space [10], rendering it suitable for ERP analysis and prediction.

**Figure 3:**
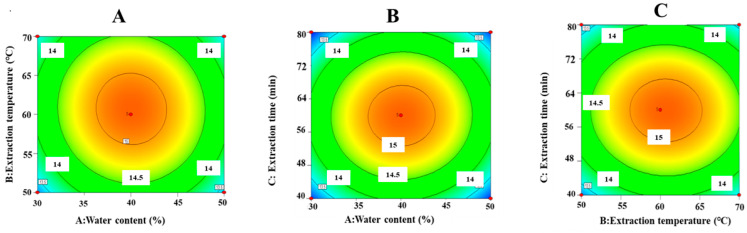
2D contour plots of interactive effects of (**A**) water contents and extraction temperature, (**B**) water contents and extraction time, and (**C**) extraction time and extraction temperature. The closer it gets to the central area (Orange-red), the higher the extraction rate.

Figure 3 displays a 2D contour plot that illustrates the interactions among various factors. A relatively flat surface and contours that approximate a circular shape suggest non-significant interactions among the factors [24,25]. The contour plots of factor interactions exhibit a near-circular shape (Figure 3), suggesting that the interactions among these factors are not significant and do not substantially affect EPR. The *p*-values for AB, AC, and BC exceed 0.05 (Table 3), further confirming that these interactions do not significantly affect EPR. By deriving and calculating the optimal values for the constructed model, the optimal process for the ultrasonic-assisted DES extraction of tea polysaccharides was identified as follows: water content of 40.05%, temperature of 60.73 °C, and extraction time of 59.91 min. To facilitate practical implementation, the optimal process was adjusted to a water content of 40%, a temperature of 61 °C, and an extraction time of 60 min.

### 3.4. Comparison of UADESTP and HWTP

**Figure 4:**
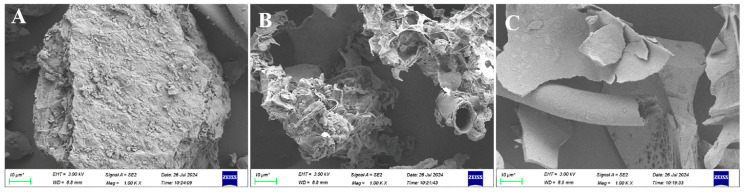
SEM of (**A**) tea powder, and tea powder extracted using (**B**) hot water and (**C**) UADES.

Under the optimal conditions, EPR was 15.89 ± 0.13%, significantly higher than that of traditional HWE (4.41 ± 0.08%) (Table 4). HWE is currently the most widely used method for plant polysaccharide extraction in industrial practice. Consequently, its extraction yield typically serves as a benchmark for evaluating the efficiency of novel extraction techniques. Generally, the extraction rates of various tea polysaccharides using HW do not exceed 5.5% [3,12], further confirming that UADES effectively enhances the extraction rate of tea polysaccharides. Similarly, UADES significantly improved the polysaccharide extraction efficiency from bamboo leaves [22]. Figure 4 shows the microstructure of tea powder before and after extraction. The unextracted tea powder exhibits a complete and compact block structure (Figure 4A). The structure is disrupted following HWE, leading to the formation of numerous cavities (Figure 4B). After UADES extraction, the structure is entirely destroyed, resulting in a flake-like morphology. These microstructural changes provide a clearer understanding of the enhanced extraction efficiency associated with UADES extraction. The total polysaccharides content of UADESTP was comparable to that of HWTP (75.47 ± 1.35% vs. 74.08± 2.51%) (Table 4). Meanwhile, UADESTPs contain more uronic acid (8.35 ± 0.26%) and less protein (12.91%) than HWTP (Table 4).

### 3.5. Characterization of UADESTP

#### 3.5.1. Mw and Monosaccharide Composition

**Figure 5:**
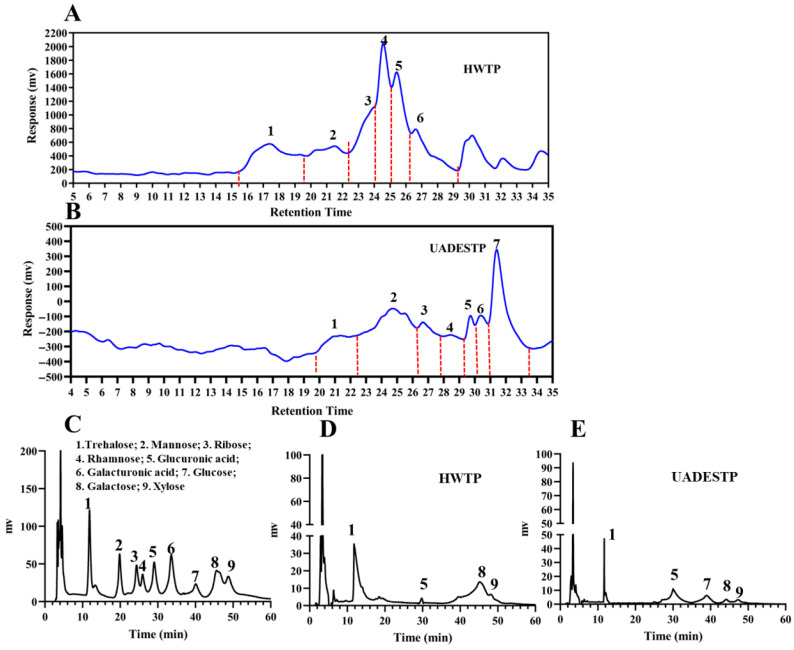
GPC profiles of (**A**) HWTP and (**B**) UADESTP. HPLC profiles of the mixture of monosaccharide standard (**C**), HWTP (**D**) and (**E**) UADESTP.

The HPGPC profile shows that HWTP comprises seven components with distinct Mws, whereas UADESTP consists of six components with varying Mws (Figure 5A,B). Lotus leaves polysaccharides extracted using DES also contain fewer components [3,26], indicating the targeted extraction by DES. Additionally, approximately 88.87% of the components in UADESTP have a Mw below 1.80 × 10^3^ Da, which is significantly lower than that of HWTP, where the majority of components exceed a Mw of 1.62 × 10^3^ Da (Table 5). Ultrasonic treatment can disrupt sensitive chemical bonds in polysaccharides and serves as a crucial method for polysaccharide degradation [5], which significantly contributes to the lower Mw of UADESTP compared to HWTP.

The HPLC results reveal a significant difference in the monosaccharide composition between UADESTP and HWTP (Figure 5C,D). HWTP primarily comprises trehalose, glucuronic acid, galactose, and xylose, with molar ratios of 32:4:36:1; in contrast, UADESTP contains trehalose, glucuronic acid, galactose, xylose, and glucose, with molar ratios of 8:16:1:10 (Table 6). These findings suggest that the monosaccharide composition of polysaccharides varies with the extraction method. A significant decrease in the proportions of galactose and trehalose was observed in UADESTP. It is postulated that ultrasound or the DES system may induce the degradation of the two monosaccharides, which aligns with other studies [27]. While glucose content in tea polysaccharides is minimally affected by extraction methods, the result herein showed a substantial elevation in glucose proportion within UADESTP [27]. The underlying mechanisms warrant further investigation. The proportion of uronic acids in UADESTP is higher, consistent with the uronic acid content results presented in Table 4. The results indicate potential differences in the bioactivity of UADESTP and HWTP.

#### 3.5.2. Microstructure

**Figure 6:**
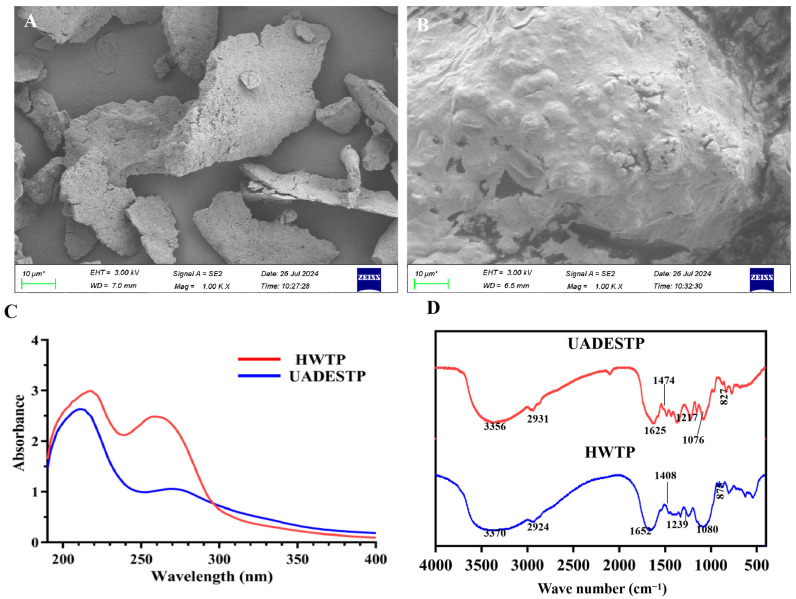
SEM of (**A**) HWTP and (**B**) UADESTP. (**C**) UV scanning and (**D**) FI-IR spectra of HWTP and UADESTP.

The microstructures of UADESTP and HWTP were analyzed. HWTP exhibits an irregular block structure characterized by micropores on its surface (Figure 6A), whereas UADESTP is composed of multiple planar, sheet-like structures adorned with numerous protrusions (Figure 6A). This structural difference is likely attributable to the distinct extraction methods used. The microstructure of the polysaccharides significantly influences their physiological activity; those with porous and sheet-like structures possess a larger specific surface area, which enhances the exposure of their active sites, thereby improving their activity [28]. Consequently, it can be inferred that there may also be notable differences in the activities of UADESTP and HWTP.

#### 3.5.3. UV and FT-IR

Figure 6C illustrates that UADESTP and HWTP display distinct absorption peaks in the range of 210–270 nm, suggesting that both polysaccharides are glycoproteins. These findings are consistent with previous reports [3]. The intensity of the absorption peak for HWTP is higher, further confirming its greater protein content (Table 4).

The FT-IR spectra of UADESTP and HWTP exhibit notable similarities (Figure 6D). The absorption at 3356/3370 cm^−1^ is caused by O–H [20]; the peak at 2931/2924 cm^−1^ is associated with the vibration of C–H [20]; and the band at 1625/1652 cm^−1^ is assigned with –COOH [22]; The peak at 1474/1408 cm^−1^ is caused by uronic acids [22]; the strong band at 1076/1080 cm^−1^ is attributed to C–OH and C–O–C from the pyran ring [10]; and the small peak at 827/875 cm^−1^ indicates the presence of β-glycosidic linkages [10].

### 3.6. Anti-Oxidant Activity of UADESTP

#### 3.6.1. Free Radical Scavenging and Reduction Abilities of UADESTP

**Figure 7:**
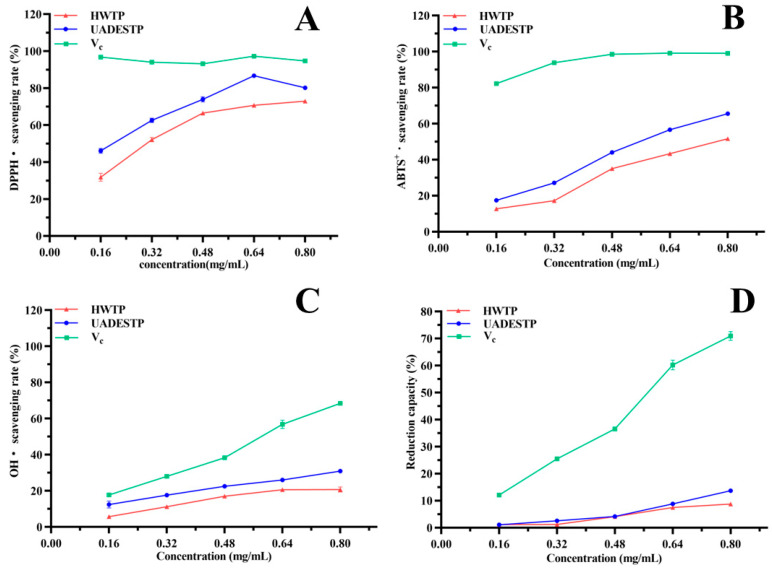
(**A**) DPPH, (**B**) ABTS^+^, (**C**) OH· scavenging rate and (**D**) reduction capability of HWTP and UADESTP.

Figure 7 shows the antioxidant ability of UADESTP and HWTP. As illustrated in Figure 7A, the scavenging rates of DPPH· by UADESTP and HWTP increased gradually with increasing concentrations, reaching maximum values of 86.69 ± 0.80% and 72.98 ± 0.69%, respectively. Moreover, the scavenging rate of DPPH· by UADESTP was significantly greater than that of HWTP (*p* < 0.05). Similarly, the scavenging rates of ABST + · and OH· by UADESTP and HWTP demonstrated concentration dependence, with UADESTP exhibiting a significantly higher scavenging effect than HWTP (*p* < 0.05) (Figure 7B,C). In the reduction capacity study, UADESTP (0.8 mg/mL) demonstrated significantly greater reduction capacity than HWTP (*p* < 0.05) (Figure 7D). The data illustrate that UADESTP exhibits more potent anti-oxidant activity than HWTP. Polysaccharides possess a substantial number of hydroxyl groups, which can donate sufficient electrons to free radicals [10]. Consequently, polysaccharides with a higher concentration exhibit more potent capacities for free radical scavenging. The Mw of polysaccharides significantly influences their antioxidant activity [29]. Polysaccharides with lower Mws demonstrate higher solubility, facilitating improved interactions with free radicals and thereby enhancing their scavenging ability [30]. Furthermore, polysaccharides with elevated uronic acid content exhibit enhanced antioxidant activity [30]. Compared to HWTP, UADESTP possesses a lower Mw and more uronic acid, both of which are critical factors contributing to its robust antioxidant activity.

#### 3.6.2. UADESTP Attenuates H_2_O_2_-Induced Oxidative Injury

**Figure 8:**
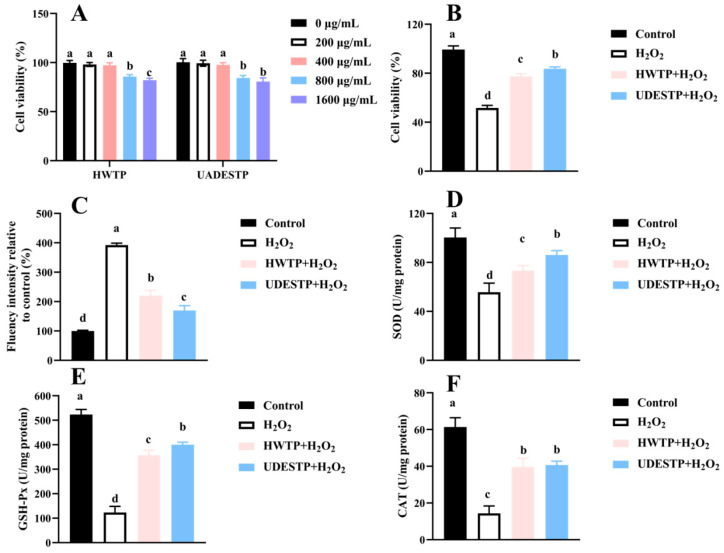
(**A**) Effect of different concentrations on the cell viability of HepG2 cells treated with various amounts of HWTP and UADESTP. Alleviating effect of HWTP and UADESTP on the H_2_O_2_-induced injury of HepG2 cells’ (**B**) cell viability, (**C**) ROS production, and activity of (**D**) SOD, (**E**) GSH-Px and (**F**) CAT. Different letters indicate significant differences (*p* < 0.05).

An oxidative-injured model was established using HepG2 cells to further evaluate the alleviating effect of tea polysaccharides on oxidative damage. Figure 8A indicates that both samples at concentrations ranging from 200 to 400 μg/mL had no influence on cell viability; therefore, 200 μg/mL was chosen in the following study. In the H_2_O_2_ group, HepG2 cells viability was significantly reduced, with a cell viability of only 51.67 ± 2.08% (Figure 8B). Pre-treatment with tea polysaccharides effectively mitigated H_2_O_2_-induced damage to the cells, with UADESTP exhibiting a more pronounced effect, restoring cell viability to 83.33 ± 1.52% (Figure 8B). Upon entering the cells, H_2_O_2_ generates excessive ROS, resulting in oxidative stress. Figure 8C illustrates that the intracellular ROS fluorescence intensity increased to approximately four times that of the control group following H_2_O_2_ treatment. In contrast, pre-treatment with tea polysaccharides significantly reduced ROS fluorescence intensity (*p* < 0.05), with the UADESTP group’s ROS levels approaching those of the control group, indicating the stronger antioxidant activity of UADESTP. Under normal physiological conditions, the endogenous antioxidant enzyme system, comprising SOD, GSH-Px, and CAT, is responsible for eliminating excess free radicals and maintaining the cellular redox balance [15]. As demonstrated in Figure 8D–F, excessive H_2_O_2_ treatment significantly diminished the activity of these enzymes, indicating that the endogenous antioxidant system was compromised. However, following pre-treatment with tea polysaccharides, the activities of SOD, GSH-Px, and CAT were significantly restored (*p* < 0.05), further indicating that tea polysaccharides can mitigate the oxidative damage induced by H_2_O_2_ in the cells. Additionally, UADESTP exhibited superior antioxidant effects, which was confirmed by the higher activities of SOD and GSH-Px in the UADESTP group. Previous studies have also demonstrated that tea polysaccharides are capable of alleviating oxidative damage in PC12 cells and oxidative stress on the liver, including restoring PC12 cell viability and endogenous oxidase activity in the liver [31]. Yan also confirmed that green tea polysaccharides (100 μg/mL) could reduce oxidative damage and apoptosis induced by H_2_O_2_ in retinal endothelial cells [32]. These studies further corroborate our results. In conclusion, compared with HWTP, UADESTPs have a better alleviating effect on oxidative damage.

## 4. Conclusions

In the present study, optimal UADES extraction conditions for tea polysaccharides were determined as follows: extraction temperature 61 °C, ultrasonic power 480 W, and extraction time 60 min. The optimal DES comprised choline chloride (CC) and ethylene glycol (EG) in a molar ratio of 1:3 with 40% water content. Compared to HWTP, UADESTP exhibited distinct monosaccharide composition, Mw, and microstructures, along with significantly enhanced antioxidant capacity. These findings facilitate the development of highly bioactive tea polysaccharides. However, practical applications remain unexplored, warranting further investigation to advance this technology.

## Figures and Tables

**Table 1:** Composition of different type of EDS.

Abbreviation	Combination	Mole Ratio	Water Content (%)
CCB	Choline chloride:1,4-butanediol	4:1	30
CCEG	Choline chloride:Ethylene glycol	2:1	30
CCG	Choline chloride:Glycerol	2:1	30
CCLA	Choline chloride:Lactic acid	1:1	30
CCOA	Choline chloride:Oxalic acid	1:1	30

**Table 2:** BBD test design and results.

Number	A (Water Content/%)	B (Temperature/°C)	C (Time/min)	ERP (%)
1	40	60	60	15.04
2	40	60	60	14.87
3	30	60	80	13.06
4	40	60	60	15.38
5	50	60	80	13.15
6	40	50	80	13.19
7	50	70	60	13.51
8	40	70	80	13.27
9	40	70	40	13.55
10	40	60	60	15.17
11	50	50	60	13.33
12	40	50	40	13.36
13	30	50	60	13.19
14	30	70	60	13.56
15	50	60	40	12.89
16	30	60	40	12.94
17	40	60	60	15.27

**Table 3:** Variance analysis of the regression model.

Source	Sum of Squares	df	Mean Square	F-Value	*p*-Value
Model	13.14	9	1.46	39.29	<0.0001
A	1.823 × 10^−3^	1	1.823 × 10^−3^	0.049	0.8310
B	0.084	1	0.084	2.26	0.1768
C	5.426 × 10^−4^	1	5.426 × 10^−4^	0.015	0.9072
AB	8.927 × 10^−3^	1	8.927 × 10^−3^	0.24	0.6390
AC	5.327 × 10^−3^	1	5.327 × 10^−3^	0.14	0.7162
BC	2.915 × 10^−3^	1	2.915 × 10^−3^	0.079	0.7862
A^2^	4.54	1	4.54	122.29	<0.0001
B^2^	2.12	1	2.12	57.14	0.0001
C^2^	5.05	1	5.05	135.90	<0.0001
Residual	0.26	7	0.037		
Lack of fit	0.099	3	0.033	0.81	0.5490
Pure error	0.16	4	0.040		
Cor Total	13.40	16			
R^2^ = 0.9806, R_adjusted_ = 0.9556, C.V. = 1.40					

**Table 4:** EPR, total polysaccharides, proteins, and uronic acid contents of tea polysaccharides extracted using different methods.

	HWE	UADES
EPR (%)	4.41± 0.08% ^b^	15.89 ± 0.13% ^a^
Total polysaccharides (%)	74.08± 2.51% ^a^	75.47 ± 1.35% ^a^
Protein (%)	20.99 ± 2.11% ^a^	12.91 ± 4.58% ^b^
Uronic acid (%)	7.22 ± 0.19 % ^b^	8.35 ± 0.26% ^a^

Note: Different letters in the same row indicate significant difference (*p* < 0.05).

**Table 5:** Mw and mass fraction of each component of HWTP and UADESTP.

Polysaccharides	Item	Component 1	Component 2	Component 3	Component 4	Component 5	Component 6	Component 7
HWTP	Mw (Da)	2.84 × 10^5^	1.52 × 10^4^	3.20 × 10^3^	1.62 × 10^3^	986	539	-
	Mass fraction (%)	17.25	11.76	15.72	24.50	20.47	10.29	-
UADESTP	Mw (Da)	1.19 × 10^4^	1.80 × 10^3^	555	322	229	190	149
	Mass fraction (%)	11.13	33.38	10.28	4.81	5.19	7.24	27.97

**Table 6:** Monosaccharide composition mole ratio of HWTP and UADESTP.

Monosaccharide	Ratio of HWTP	Ratio of UADESTP
Trehalose	32	8
Glucuronic acid	4	16
Galactose	36	1
Xylose	1	1
Glucose,	-	10

Note: “-” stands for not detected.

## Data Availability

The original contributions presented in this study are included in the article. Further inquiries can be directed to the corresponding author.

## Abbreviations

The following abbreviations are used in this manuscript:

BBD	Box–Behnken design
CAT	catalase
CC	choline chloride
ERP	extraction rate of polysaccharide
EG	ethylene glycol
FT-IR	Fourier transform infrared spectroscopy
GSH-Px	glutathione peroxidase
FBS	Fetal bovine serum
HPGPC	high-performance gel permeation chromatography
HW	hot water
HBD	hydrogen bond donors
HWTP	hot water extracted tea polysaccharides
HBA	hydrogen bond acceptors
HPLC	high-performance liquid chromatography
LA	lactic acid
OA	oxalic acid
SOD	superoxide dismutase
SEM	Scanning electron microscope
UADESTP	ultrasonic-assisted DES extracted tea polysaccharides
ROS	reactive oxygen species
UADES	ultrasonic-assisted deep eutectic solvents
